# Differences in genetic variation in antigen-processing machinery components and association with cervical carcinoma risk in two Indonesian populations

**DOI:** 10.1007/s00251-015-0834-5

**Published:** 2015-03-22

**Authors:** Akash M. Mehta, Vivian M. Spaans, Nyoman Bayu Mahendra, Elisabeth M. Osse, Jessica N. I. Vet, Gatot Purwoto, I. G. D. Surya, Santoso Cornian, Alexander A. Peters, Gert J. Fleuren, Ekaterina S. Jordanova

**Affiliations:** 1 Department of Surgical Oncology, The Netherlands Cancer Institute - Antoni van Leeuwenhoek Hospital, Amsterdam, The Netherlands; 2 Department of Pathology, Leiden University Medical Center, Leiden, The Netherlands; 3 Department of Gynecology, Leiden University Medical Center, Leiden, The Netherlands; 4 Department of Gynecology and Obstetrics, Prima Medika Hospital, Denpasar, Bali Indonesia; 5 Department of Gynecology, Universitas Indonesia, Jakarta, Indonesia; 6 Department of Gynecology, Universitas Udayana, Bali, Indonesia; 7 Department of Anatomy, Universitas Indonesia, Jakarta, Indonesia

**Keywords:** Cervical carcinoma, Single nucleotide polymorphisms, Human papilloma virus, Indonesia, Haplotype interaction, Antigen-processing machinery, Genetic variation

## Abstract

Genetic variation of antigen-processing machinery (APM) components has been shown to be associated with cervical carcinoma risk and outcome in a genetically homogeneous Dutch population. However, the role of APM component single nucleotide polymorphisms (SNPs) in genetically heterogeneous populations with different distributions of human papillomavirus (HPV) subtypes remains unclear. Eleven non-synonymous, coding SNPs in the *TAP1*, *TAP2*, *LMP2*, *LMP7* and *ERAP1* genes were genotyped in cervical carcinoma patients and healthy controls from two distinct Indonesian populations (Balinese and Javanese). Individual genotype and allele distributions were investigated using single-marker analysis, and combined SNP effects were assessed by haplotype construction and haplotype interaction analysis. Allele distribution patterns in Bali and Java differed in relation to cervical carcinoma risk, with four *ERAP1* SNPs and one *TAP2* SNP in the Javanese population showing significant association with cervical carcinoma risk, while in the Balinese population, only one *TAP2* SNP showed this association. Multimarker analysis demonstrated that in the Javanese patients, one specific haplotype, consisting of the *ERAP1*-*575* locus on chromosome 5 and the *TAP2*-*379* and *TAP2-651* loci on chromosome 6, was significantly associated with cervical carcinoma risk (global *P* = 0.008); no significant haplotype associations were found in the Balinese population. These data indicate not only that genetic variation in APM component genes is associated with cervical carcinoma risk in Indonesia but also that the patterns of association differ depending on background genetic composition and possibly on differences in HPV type distribution.

## Introduction

The antigen-processing machinery (APM) comprises various components working together to determine the presentation of peptides derived from intracellular proteins by human leucocyte antigen (HLA) class I molecules (Heemels and Ploegh 1995). The APM has been shown to be important in immune recognition of virally infected cells and tumour cells. Defects in the APM have been shown to be associated with progression and impaired survival in various tumours (Facoetti et al. 2005; Meissner et al. 2005; Seliger et al. 2006). In particular, we have shown previously that downregulation of endoplasmic reticulum aminopeptidase associated with antigen presentation 1 (ERAP1), transporter associated with antigen presentation 1 (TAP1) and low molecular weight peptide 7 (LMP7) is significantly associated with decreased survival in human papilloma virus (HPV) associated cervical carcinoma (Mehta et al. 2008).

Apart from APM protein expression defects, we have previously hypothesized that genetic variation in the genes encoding APM components may contribute to susceptibility to cervical carcinoma. We have demonstrated that various single nucleotide polymorphisms (SNPs) in the *LMP7*, *TAP2* and *ERAP1* genes were significantly associated with risk of developing cervical carcinoma in the Dutch population (Mehta et al. 2007). Moreover, two *ERAP1* SNPs were found to be significantly associated with poor survival in Dutch cervical cancer patients (Mehta et al. 2009).

Although these data suggest that genetic variation in APM genes is an important determinant in the development and progression of cervical carcinoma in the Dutch population, it remains to be determined whether these findings can be extrapolated to other gene pools and populations. Our group has previously shown that the distribution of HPV subtypes among cervical carcinoma patients in Indonesia, where high incidence of HPV infection has been recorded, differs from other geographical areas (Vet et al. 2008). This was hypothesized to be caused by the relative isolation of certain Indonesian regions and subpopulations.

To ascertain the extent to which APM genetic variation may contribute to cervical carcinoma risk in regions in which HPV is virtually endemic, we have studied the genotypes and genotype interactions of a previously reported set of SNPs in distinct Indonesian populations from Java and Bali. We demonstrate that, as in the Dutch population, APM genetic variation is associated with cervical carcinoma risk but that the association patterns are different in the two Indonesian populations.

## Methods

### Subjects

Cervical cancer cases and healthy controls were derived from two distinct Indonesian populations from Java and Bali. In the Javanese population, cervical carcinoma cases consisted of 98 female patients with histologically confirmed invasive cervical cancer from the regions of Jakarta and Tasikmalaya, while 105 healthy female controls from the same area were included in the study. In the Balinese population, 103 cervical carcinoma patients and 68 healthy controls were included.

HPV typing was performed by using the SPF10 primer set and INNO-LIPA HPV Genotyping Extra line probe assay (Fujirebio Europe, Ghent, Belgium). In 87 (89 %) of the Javanese patients, high-risk HPV was detected: The most common types were HPV16 (*n* = 35; 35.7 %), HPV18 (*n* = 28; 28.6 %) and HPV52 (*n* = 15; 15.3 %). In 77 (74.8 %) of the 103 Balinese patients, high-risk HV was detected, of whom 38 (36.9 %) were HPV16 positive, 16 (15.5 %) were HPV18 positive and 8 (7.8 %) were HPV52 positive.

DNA isolation from cervical swabs was performed by Baseclear (Leiden, the Netherlands) using a magnetic bead-based assay. DNA was extracted from formalin-fixed, paraffin-embedded cervical tumour tissue using the Tissue Preparation System (Siemens Healthcare Diagnostics, NY, USA) (Hennig et al. 2010).

### SNP genotyping

Eleven previously documented polymorphisms in the LMP2, LMP7, TAP1, TAP2 (chromosome 6) and ERAP1 (chromosome 5) coding regions were genotyped using TaqMan SNP Genotyping Assays (Applied Biosystems, Inc., Foster City, CA) based on the 5′-nuclease allelic discrimination assay as described previously (Livak 1999). A combination of pre-designed assays (available as Assays-on-Demand products) and custom SNP Genotyping Assays (available through the Assays-by-Design service) was used. Probe and primer sequences for the Custom SNP Genotyping Assays and available information for the pre-designed SNP Genotyping Assays have been described previously (Mehta et al. 2007).

SNP genotyping was performed as described previously (Mehta et al. 2007). In short, real-time PCR was performed in a 5 ul reaction volume containing 10 ng of patient or control genomic DNA (dried by overnight evaporation), 1× final concentration of TaqMan Universal PCR Mix and 1× final concentration of the appropriate TaqMan SNP Genotyping Assay (0.9 umol/l of each primer and 0.2 umol/l of each probe). All liquid handling was performed with a Genesis RSP 100 automated liquid pipetting system (Tecan Group Ltd., Männedorf, Switzerland). PCR reactions were carried out and read in an ABI PRISM 7900HT sequence detection system (Applied Biosystems) with the following cycling conditions: 95 °C for 10 min, followed by 40 cycles of 92 °C for 15 s and 60 °C for 1 min. Data acquisition and allelic discrimination were performed using Sequence Detection Systems software version 2.2.2 (Applied Biosystems).

### Statistical analysis

For each polymorphism, deviation of the genotype frequencies from those expected under Hardy-Weinberg equilibrium was assessed using chi-squared tests implemented in Haploview software version 3.2 (Barrett et al. 2005). Distribution of possible genotypes at the investigated SNP loci was compared among cases and controls using the chi-squared test. The Armitage trend test, as implemented in FAMHAP software version 19, was used to test for a trend in cervical carcinoma risk with individual allele numbers (Sasieni 1997).

Chromosome 5 and 6 haplotypes were constructed, haplotype frequencies were estimated, and haplotype interaction analysis of the two chromosomes was performed using the expectation-maximization (EM) algorithm implemented in FAMHAP software version 19 (Becker et al. 2005a, b; Becker and Knapp 2004; Mehta et al. 2007). Miettinen’s formula was used to calculate the population attributable fraction (PAF), i.e. the fraction of all cervical carcinoma cases attributable to a haplotype observed to be significantly associated with disease (Hanley 2001; Miettinen 1974).

All tests were two-sided, and the significance level was set to 0.05. Where appropriate, *P* values were corrected for multiple testing. Statistical analyses were performed with IBM SPSS Statistics software version 20 (SPSS Inc., Chicago, IL).

## Results

### Genotype and allele distributions

Eleven previously reported non-synonymous polymorphisms in the coding regions of *LMP2*, *LMP7*, *TAP1*, *TAP2* and *ERAP1* were tested separately in the Javanese and Balinese populations (Mehta et al. 2007, 2009). Major and minor alleles for the tested loci are shown in Table 1. In both populations, genotype distributions in cases and controls were in Hardy-Weinberg equilibrium (data not shown). The results of univariate analyses of the various genotype frequencies among cases and controls, and the results of the Armitage trend test, are shown in Tables 2 and 3 for the Javanese and Balinese populations, respectively. As shown, in the Javanese population, four *ERAP1* SNPs and the *TAP2*-651 SNP were significantly associated with cervical carcinoma occurrence, whereas in the Balinese population, only the *TAP2*-651 locus showed this association.

**Table 1:** Single nucleotide polymorphism (SNP) sequence variation

SNP ID (dbSNP reference)	Nucleotide variation^a^
*ERAP1-56* (rs3734016)	c.166G > A
*ERAP1-127* (rs26653)	c.380G > C
*ERAP1-276* (rs26618)	c.828 T > C
*ERAP1-528* (rs30187)	c.1583C > T
*ERAP1-575* (rs10050860)	c.1723C > T
*ERAP1-730* (rs27044)	c.2188C > G
*TAP1-333* (rs4148880)	c.1177A > G
*TAP2-379* (rs4148873)	c.1135G > A
*TAP2-651* (rs4148876)	c.1951C > A
*LMP2-60* (rs9276814)	c.179G > A
*LMP7-145* (rs2071543)	c.145C > A

^a^Genbank accession numbers for reference sequences: NM_016442.2 for *ERAP1*, NM_000593.5 for *TAP1*, NM_000544.3 for *TAP2*, NM_00280.4 for *LMP2*, and NM_148919.3 for *LMP7*

**Table 2:** SNP genotype distributions in the Javanese population

SNP	Controls (*n* = 105) *n* (%)			Cases (*n* = 98) *n* (%)			Genotype distribution *P* value	Armitage *P* value
	Major allele homozygotes	Heterozygotes	Minor allele homozygotes	Major allele homozygotes	Heterozygotes	Minor allele homozygotes		
*ERAP1-56*	85 (83.3)	3 (2.9)	14 (13.7)	73 (77.7)	1 (1.1)	20 (21.3)	0.266	0.224
*ERAP1-127*	25 (25.0)	49 (49.0)	26 (26.0)	34 (37.4)	41 (45.1)	16 (17.6)	0.132	**0.047**
*ERAP1-276*	39 (37.1)	51 (48.6)	15 (14.3)	31 (34.4)	40 (44.4)	19 (21.1)	0.456	0.348
*ERAP1-528*	25 (24.5)	49 (48.0)	28 (27.5)	32 (35.6)	43 (47.8)	15 (16.7)	0.108	**0.036**
*ERAP1-575*	100 (96.2)	4 (3.8)	0 (0.0)	84 (88.4)	11 (11.6)	0 (0.0)	0.119	**0.039**
*ERAP1-730*	26 (25.0)	53 (51.0)	25 (24.0)	35 (37.6)	44 (47.3)	14 (15.1)	0.097	**0.031**
*TAP1-333*	45 (44.1)	46 (45.1)	11 (10.8)	40 (46.0)	43 (49.4)	4 (4.6)	0.288	0.379
*TAP2-379*	44 (43.6)	44 (43.6)	13 (12.9)	48 (52.7)	34 (37.4)	9 (9.9)	0.435	0.215
*TAP2-651*	97 (93.3)	7 (6.7)	0 (0.0)	94 (98.9)	1 (1.1)	0 (0.0)	0.126	**0.042**
*LMP2-60*	55 (53.4)	42 (40.8)	6 (5.8)	47 (51.1)	40 (43.5)	5 (5.4)	0.929	0.824
*LMP7-145*	84 (81.6)	18 (17.5)	1 (1.0)	84 (90.3)	9 (9.7)	0 (0.0)	0.174	0.065

*n (%)* number (percentage)

All *P* values are corrected for multiple testing

Bold *P* values indicate statistical significance, i.e. *P* < 0.05

**Table 3:** SNP genotype distributions in the Balinese population

SNP	Controls (*n* = 68) *n* (%)			Cases (*n* = 103) *n* (%)			Genotype distribution *P* value	Armitage *P* value
	Major allele homozygotes	Heterozygotes	Minor allele homozygotes	Major allele homozygotes	Heterozygotes	Minor allele homozygotes		
*ERAP1-56*	35 (85.4)	0 (0.0)	6 (14.6)	13 (72.2)	1 (5.6)	4 (22.2)	0.227	0.331
*ERAP1-127*	21 (33.9)	36 (8.1)	5 (8.1)	35 (35.4)	49 (49.5)	15 (15.2)	0.351	0.594
*ERAP1-276*	13 (30.2)	26 (60.5)	4 (9.3)	7 (35.0)	10 (50.0)	3 (15.0)	0.684	0.956
*ERAP1-528*	10 (23.8)	28 (66.7)	4 (9.5)	5 (25.0)	12 (60.0)	3 (15.0)	0.794	0.786
*ERAP1-575*	38 (88.4)	5 (11.6)	0 (0.0)	18 (90.0)	2 (10.0)	0 (0.0)	0.982	0.848
*ERAP1-730*	22 (35.5)	36 (58.1)	4 (6.5)	39 (39.0)	47 (47.0)	14 (14.0)	0.223	0.699
*TAP1-333*	27 (45.0)	25 (41.7)	8 (13.3)	53 (54.1)	29 (29.6)	16 (16.3)	0.299	0.611
*TAP2-379*	26 (60.5)	15 (34.9)	2 (4.7)	12 (63.2)	5 (26.3)	2 (10.5)	0.600	0.841
*TAP2-651*	41 (66.1)	3 (4.8)	18 (29.0)	35 (35.7)	1 (1.0)	62 (63.3)	<0.001	**<0.001**
*LMP2-60*	24 (38.1)	35 (55.6)	4 (6.3)	35 (34.7)	50 (49.5)	16 (15.8)	0.195	0.217
*LMP7-145*	57 (93.4)	4 (6.6)	0 (0.0)	89 (89.9)	9 (9.1)	1 (1.0)	0.617	0.370

*n (%)* number (percentage)

All *P* values are corrected for multiple testing

Bold *P* values indicate statistical significance, i.e. *P* < 0.05

Single allele distributions were determined in both groups to further evaluate the effect of individual major and minor alleles at each of the 11 loci. As shown in Table 4, in the Javanese population, allele distributions at the *ERAP1-127*, *ERAP1-528* and *ERAP1-730* loci were significantly associated with altered cervical carcinoma risk (*P* = 0.042, 0.032 and 0.031, respectively); presence of the minor allele at these loci was associated with decreased cancer risk.

**Table 4:** SNP allele distributions in the Javanese population

SNP	Controls (*n* = 210) *n* (%)		Cases (*n* = 196) *n* (%)		OR (95 % CI) (minor vs*.* major allele)	Allele distribution *P* value
	Major allele	Minor allele	Major allele	Minor allele		
*ERAP1-56*	173 (84.8)	31 (15.2)	147 (78.2)	41 (21.8)	1.557 (0.929–2.607)	0.092
*ERAP1-127*	99 (49.5)	101 (50.5)	109 (59.9)	73 (40.1)	0.656 (0.437–0.985)	**0.042**
*ERAP1-276*	129 (61.4)	81 (38.6)	102 (56.7)	78 (43.3)	1.218 (0.812–1.826)	0.346
*ERAP1-528*	99 (48.5)	105 (51.5)	107 (59.4)	73 (40.6)	0.643 (0.429–0.964)	**0.032**
*ERAP1-575*	204 (98.1)	4 (1.9)	179 (94.2)	11 (5.8)	3.134 (0.981–10.016)	0.054
*ERAP1-730*	105 (50.5)	103 (49.5)	114 (61.3)	72 (38.7)	0.644 (0.431–0.962)	**0.031**
*TAP1-333*	136 (66.7)	68 (33.3)	123 (70.7)	51 (29.3)	0.829 (0.535–1.284)	0.408
*TAP2-379*	132 (65.3)	70 (34.7)	130 (71.4)	52 (28.6)	0.754 (0.489–1.163)	0.203
*TAP2-651*	201 (96.6)	7 (3.4)	189 (99.5)	1 (0.5)	0.152 (0.019–1.247)	0.077
*LMP2-60*	152 (73.8)	54 (26.2)	134 (72.8)	50 (27.2)	1.050 (0.670–1.646)	0.843
*LMP7-145*	186 (90.3)	20 (9.7)	177 (95.2)	9 (4.8)	0.473 (0.210–1.066)	0.071

*n (%)* number (percentage), *OR* odds ratio, *95 % CI* 95 % confidence interval

All *P* values are corrected for multiple testing

Bold *P* values indicate statistical significance, i.e. *P* < 0.05

As shown in Table 5, in the Balinese population, only the *TAP2-651* locus was significantly associated with cervical carcinoma risk, with the minor allele increasing this risk (*P* < 0.001).

**Table 5:** SNP allele distributions in the Balinese population

SNP	Controls (*n* = 136) *n* (%)		Cases (*n* = 206) *n* (%)		OR (95 % CI) (minor vs. major allele)	Allele distribution *P* value
	Major allele	Minor allele	Major allele	Minor allele		
*ERAP1-56*	70 (85.4)	12 (14.6)	27 (75.0)	9 (25.0)	1.944 (0.736–5.138)	0.181
*ERAP1-127*	78 (62.9)	46 (37.1)	119 (60.1)	79 (39.9)	1.126 (0.709–1.787)	0.627
*ERAP1-276*	52 (60.5)	34 (39.5)	24 (60.0)	16 (40.0)	1.020 (0.474–2.194)	0.963
*ERAP1-528*	48 (57.1)	36 (42.9)	22 (55.0)	18 (45.0)	1.091 (0.511–2.328)	0.833
*ERAP1-575*	81 (94.2)	5 (5.8)	38 (95.0)	2 (5.0)	0.853 (0.158–4.596)	0.863
*ERAP1-730*	80 (64.5)	44 (35.5)	125 (62.5)	75 (37.5)	1.091 (0.684–1.739)	0.560
*TAP1-333*	79 (65.8)	41 (34.2)	135 (68.9)	61 (31.1)	0.871 (0.537–1.412)	0.587
*TAP2-379*	67 (77.9)	19 (22.1)	29 (76.3)	9 (23.7)	1.094 (0.443–2.705)	0.856
*TAP2-651*	85 (68.5)	39 (31.5)	71 (36.2)	125 (63.8)	3.837 (2.379–6.189)	**<0.001**
*LMP2-60*	83 (65.9)	43 (34.1)	120 (59.4)	82 (40.6)	1.319 (0.830–2.096)	0.244
*LMP7-145*	118 (96.7)	4 (3.3)	187 (94.4)	11 (5.6)	1.735 (0.540–5.576)	0.361

n (%) = *n (%)* number (percentage), *OR* odds ratio, *95 % CI* 95 % confidence interval

All *P* values are corrected for multiple testing

Bold *P* values indicate statistical significance, i.e. *P* < 0.05

### Haplotype construction and frequency estimation

As our previous study demonstrated that genotype combinations contribute to cervical carcinoma development to a larger degree than individual SNPs, the effect of various SNP combinations spanning chromosomes 5 and 6 was assessed. To this end, separate haplotypes were constructed spanning the six *ERAP1* SNPs on chromosome 5 and the five *LMP2*, *LMP7*, *TAP1* and *TAP2* SNPs on chromosome 6. As there was no obvious linkage disequilibrium among the SNPs investigated (data not shown), the EM algorithm was used to estimate the most likely haplotype arrangements spanning all loci (Becker et al. 2005a, b; Becker and Knapp 2004). Only haplotypes with a frequency >1.0 % in controls were included for further analysis.

As shown in Table 6, application of the EM algorithm on the six SNPs in the *ERAP1* gene region on chromosome 5 yielded eight common haplotypes with a frequency ≥1 % in controls, representing >99 % of all possible haplotypes in the Javanese population. For the five SNPs on chromosome 6, nine common haplotypes were generated representing >99 % of all possible haplotypes in this population. As shown, in the Javanese population, no significant global differences in frequency distributions for the constructed haplotypes were found among cases and controls.

**Table 6:** Estimated haplotype frequencies based on all SNPs and tests for distributions among cases and controls in the Javanese population

Chromosome	Haplotype ID	Haplotype sequence	Controls (*n* = 210) *n* (%)	Cases (*n* = 196) *n* (%)	OR (95 % CI)	Haplotype-specific *P* value	Global *P* value
5^a^	H1	G–C–T–T–C–G	82 (42.0)	51 (31.9)	0.65 (0.42–1.00)	0.051	0.162
	H2	G–G–C–C–C–C	70 (35.5)	57 (35.6)	1.01 (0.65–1.55)	0.968	
	H3	A–G–T–C–C–C	12 (6.2)	18 (11.3)	1.93 (0.90–4.13)	0.090	
	H4	A–C–T–T–C–G	11 (5.4)	12 (7.5)	1.43 (0.61–3.37)	0.420	
	H5	A–G–C–C–C–C	7 (3.7)	9 (5.6)	1.54 (0.57–4.17)	0.402	
	H6	G–C–T–T–C–C	5 (2.6)	2 (1.3)	0.49 (0.09–2.53)	0.409	
	H7	G–G–T–C–T–C	3 (1.5)	8 (5.0)	3.39 (0.88–13.01)	0.075	
	H8	G–C–T–C–C–C	2 (1.0)	2 (1.3)	1.2 (0.17–8.50)	0.865	
6^b^	H1	G–C–G–A–G	49 (24.7)	54 (33.5)	1.54 (0.97–2.43)	0.065	0.331
	H2	G–C–G–G–G	45 (22.5)	35 (21.3)	0.93 (0.57–1.54)	0.787	
	H3	A–C–G–A–A	34 (17.1)	26 (16.1)	0.93 (0.54–1.63)	0.809	
	H4	G–A–G–A–G	17 (8.6)	8 (4.8)	0.53 (0.22–1.28)	0.158	
	H5	G–C–G–G–A	17 (8.5)	11 (6.9)	0.79 (0.36–1.73)	0.568	
	H6	G–C–G–A–A	13 (6.6)	8 (5.0)	0.75 (0.30–1.84)	0.545	
	H7	A–C–G–A–G	12 (6.2)	16 (10.1)	1.71 (0.79–3.68)	0.172	
	H8	G–C–A–A–G	3 (1.7)	1 (0.6)	0.37 (0.04–3.45)	0.389	
	H9	A–C–G–G–A	3 (1.4)	2 (0.9)	0.63 (0.09–4.61)	0.658	

^a^Chromosome 5 haplotype sequences listed in following order: *ERAP1-56*–*ERAP1-127*–*ERAP1-276*–*ERAP1-528*–*ERAP1-575*–*ERAP1-730*

^b^Chromosome 6 haplotype sequences listed in following order: *LMP2-60*–*LMP7-145*–*TAP2-651*–*TAP1-333*–*TAP2-379*

All *P* values are corrected for multiple testing

For the Balinese population, six chromosome 5 and five chromosome 6 haplotypes were generated with a frequency ≥1 % in controls, as shown in Table 7. Here too, no significant global differences in frequency distributions for the constructed haplotypes could be demonstrated among cases and controls.

**Table 7:** Estimated haplotype frequencies based on all SNPs and tests for distributions among cases and controls in the Balinese population

Chromosome	Haplotype ID	Haplotype sequence	Controls (*n* = 136) *n* (%)	Cases (*n* = 206) *n* (%)	OR (95 % CI)	Haplotype-specific *P* value	Global *P* value
5^a^	H1	G–C–T–C–C–G	30 (39.5)	11 (32.4)	0.73 (0.31–1.72)	0.481	0.848
	H2	G–G–C–T–C–C	27 (35.5)	11 (32.4)	0.87 (0.37–2.05)	0.763	
	H3	A–G–T–T–C–C	7 (9.2)	4 (11.8)	1.31 (0.36–4.83)	0.697	
	H4	G–G–T–T–T–C	5 (6.6)	2 (5.9)	0.89 (0.16–4.82)	0.902	
	H5	A–G–C–T–C–C	3 (3.9)	3 (8.8)	2.35 (0.45–12.32)	0.316	
	H6	A–C–T–C–C–G	2 (2.6)	1 (2.9)	1.12 (0.10–12.80)	0.933	
6^b^	H1	A–G–C–G–C	25 (32.5)	7 (21.6)	0.57 (0.21–1.54)	0.272	0.472
	H2	G–G–C–G–C	20 (26.2)	6 (21.4)	0.77 (0.28–2.10)	0.624	
	H3	A–G–C–A–C	13 (16.4)	6 (20.4)	1.31 (0.45–3.81)	0.633	
	H4	A–A–C–A–C	11 (14.4)	7 (23.0)	1.77 (0.61–5.09)	0.295	
	H5	A–G–C–G–A	2 (3.0)	2 (6.7)	2.35 (0.34–16.41)	0.395	

^a^Chromosome 5 haplotype sequences listed in following order: *ERAP1-56*–*ERAP1-127*–*ERAP1-276*–*ERAP1-528*–*ERAP1-575*–*ERAP1-730*

^b^Chromosome 6 haplotype sequences listed in following order: *LMP2-60*–*LMP7-145*–*TAP2-651*–*TAP1-333*–*TAP2-379*

All *P* values are corrected for multiple testing

As the perceived lack of association between the various haplotypes and cancer risk may have resulted from the high number of degrees of freedom in these distributions, permutational chi-squared testing was performed to identify specific marker combinations along which haplotypes could be grouped, thus limiting the number of degrees of freedom. This procedure did not yield any statistically significant differences in distribution in the Balinese population for either chromosome 5 or 6.

However, in the Javanese population, grouping the chromosome 5 haplotypes according to *ERAP1-575* and *ERAP1-730* genotypes yielded a statistically significant difference in distribution (*P* = 0.014), as shown in Table 8. In particular, the combination of a minor allele at *ERAP1-575* and a major allele at *ERAP1-730* was most significantly associated with increased cervical carcinoma risk, the PAF for this haplotype being 4.6 %, as calculated using Miettinen’s formula. Similarly, grouping chromosome 6 haplotypes according to the *TAP2-379* and *TAP2-651* genotypes led to a statistically significant difference in distribution (*P* = 0.042). Presence of the major allele at both loci was most significantly associated with increased cancer risk, with a PAF of 56.7 %.

**Table 8:** Chromosome 5 and 6 significant haplotype permutations in the Javanese population

Haplotype sequence	Controls (*n* = 210) *n* (%)	Cases (*n* = 196) *n* (%)	OR (95 % CI)	Haplotype-specific *P* value	Global *P* value
Chromosome 5					
*ERAP1-575*–*ERAP1-730*					
C–G	102 (49.0)	72 (39.1)	0.67 (0.45–1.00)	0.049	**0.014**
C–C	102 (49.0)	101 (54.9)	1.26 (0.85–1.88)	0.257	
T–C	3 (1.4)	11 (6.0)	4.35 (1.18–15.86)	**0.026**	
Chromosome 6					
*TAP2-379*–*TAP2-651*					
A–G	19 (9.2)	9 (4.9)	0.51 (0.22–1.15)	0.110	**0.042**
C–G	180 (87.4)	174 (94.6)	2.50 (1.17–5.35)	**0.018**	
C–A	6 (2.9)	1 (0.5)	0.18 (0.02–1.55)	0.122	

All *P* values are corrected for multiple testing

Bold *P* values indicate statistical significance, i.e. *P* < 0.05

### Haplotype interaction analysis of chromosomes 5 and 6

Haplotype interaction analysis of both chromosome regions was performed to assess the extent to which the chromosome 5 and 6 haplotype blocks may synergistically affect cervical carcinoma risk.

In the Balinese population, no haplotype combinations with a significant association with cancer risk could be identified.

However, as shown in Table 9, in the Javanese population, combined analysis of both haplotype blocks identified a specific combination consisting of the *ERAP1-575* locus on chromosome 5 and the *TAP2-379* and *TAP2-651* markers on chromosome 6 which had a significantly different distribution among cases and controls (global *P* = 0.008). Specifically, the combination of a minor allele at *ERAP1-*575 with major alleles at both *TAP2* loci was associated with an increased risk of cervical cancer; the PAF for this haplotype was 3.9 %.

**Table 9:** Haplotype interaction analysis in the Javanese population

Haplotype sequence	Controls (*n* = 210) *n* (%)	Cases (*n* = 196) *n* (%)	OR (95 % CI)	Haplotype-specific *P* value	Global *P* value
*ERAP1-575*–*TAP2-379*–*TAP2-651*					
C–A–G	19 (9.0)	8 (4.4)	0.47 (0.20–1.09)	0.081	**0.008**
C–C–G	177 (85.9)	162 (89.0)	1.33 (0.72–1.44)	0.366	
C–C–A	5 (2.4)	1 (0.5)	0.22 (0.03–1.92)	0.154	
T–C–G	4 (1.7)	10 (5.5)	3.36 (0.98–11.56)	**0.054**	

All *P* values are corrected for multiple testing

Bold *P* values indicate statistical significance, i.e. *P* < 0.05

## Discussion

The APM is considered to be a key factor in HLA class I peptide presentation, which, in turn, mediates recognition and lysis of transformed cells by cytotoxic T lymphocytes. Alterations in the APM have been shown to be associated with altered repertoires of peptides available for presentation, thereby influencing the possibility and extent of T lymphocyte-mediated immune reactions (Chang and Ferrone 2007; Falk and Rotzschke 2002; Hammer et al. 2006; Saric et al. 2002; Saveanu et al. 2005; Yan et al. 2006; York et al. 2002, 2006). Moreover, multiple associations between tumour progression and APM component defects have been described. In particular, we have previously shown that downregulation of TAP1, LMP7 and ERAP1 expression is associated with decreased survival in HPV-mediated cervical carcinoma in Dutch patients (Mehta et al. 2008).

A growing body of evidence is emerging pointing towards a genetic predisposition to HPV-mediated malignant transformation. Genetic variation in two APM SNPs has been associated with HPV-associated esophageal carcinoma (Cao et al. 2005). Furthermore, we have previously demonstrated that genetic variation in the genes encoding TAP2, LMP7 and ERAP1 is significantly associated with increased risk of developing cervical carcinoma and with poor prognosis among cervical carcinoma patients and that the effect of specific combinations of SNPs is cumulative as compared to the individual SNPs (Mehta et al. 2007, 2009). These data were limited to a relatively homogeneous Dutch population; evidence regarding such associations in other heterogeneous populations is scarce.

Therefore, in the present study, we have investigated the association between 11 previously documented, non-synonymous SNPs in seven genes encoding different APM components and cervical carcinoma risk in two distinct Indonesian populations. Our results show that genotype and allele distributions at various loci are significantly associated with occurrence of cervical carcinoma but that patterns of association differ greatly between the two populations. Specifically, in the Javanese population, a combination of the *ERAP1-575* locus on chromosome 5 and the *TAP2-379* and *TAP2-651* markers on chromosome 6 was significantly associated with cervical carcinoma risk, whereas in the Balinese population, only the *TAP2-651* locus exhibited this association.

The findings in the Javanese population underline the role of genetic variation at the *ERAP1* and *TAP2* loci in cervical carcinoma as previously described by us (Mehta et al. 2007, 2009). Genetic variation at these loci may cause functional alterations in the associated proteins, e.g. TAP transport efficiency and ERAP1 proteolytic activity. Additionally, structural stability of the associated mRNA and protein may be affected by these SNPs. Ultimately, these changes may lead to variation in the repertoire of HPV-derived peptides available for HLA class I-mediated presentation, thereby affecting immune evasion of HPV-transformed cells. However, to date, there is no conclusive evidence regarding the functional and/or structural consequences of genetic variation at these loci.

As stated previously, the observed associations between APM SNPs and cervical carcinoma may not be caused by the investigated loci at all but may reflect effects of other loci in linkage disequilibrium with the SNPs in the present study. However, to our knowledge, there is no data concerning other chromosome 5 or 6 loci in relation to cervical carcinoma risk. Moreover, the pattern we have described here and in our previous studies, i.e. that unlinked loci on different chromosomes interact in a contribution to change susceptibility to disease, is in keeping with similar findings in other vertebrates, where it has been suggested that variation in the APM pathways may be more important than variation in the major histocompatibility complex (MHC) itself in the attenuation of immune responses (Kaufman 2013).

The striking differences in patterns of association of genetic variation in the Javanese and Balinese populations may be caused by several mechanisms. Historically, Bali was a relatively isolated region of Indonesia with therefore a more homogeneous genepool. In contrast, Java has been subject to extensive migration and has a more heterogeneous genetic composition (Chow et al. 2005; Shepard et al. 2005). Moreover, our group has previously shown that the distribution of oncogenic HPV subtypes differs greatly amongst these Indonesian regions (Vet et al. 2008). Therefore, differences in the genetic variation patterns in these regions in relation to cervical carcinoma occurrence may reflect a combination of different genetic composition and differences in HPV subtype distribution. This leads to the hypothesis that different HPV subtypes may exploit different host genetic factors to evade the immune system and establish malignant transformation. This may also explain the difference in association patterns in the current study as compared to the Dutch population, which we have described previously (Mehta et al. 2007). Moreover, the genetic homogeneity of the Balinese population may explain why a single locus exhibits such a strong association with cervical carcinoma risk as opposed to a combination of loci in the genetically heterogeneous Javanese population.

In conclusion, we report the systematic analysis of several SNPs in genes encoding APM components in cervical carcinoma in two distinct Indonesian populations. In this analysis, we utilize a previously described robust and comprehensive statistical method for evaluation of multimarker effects and of haplotype interactions. We demonstrate a significant association between genetic variation in specific combinations of these SNPs and cervical carcinoma risk and, more importantly, that these association patterns differ between the two populations. We hypothesize that these differences reflect not only different genetic compositions of the populations but also well-documented differences in HPV subtype distributions. This insight, combined with ongoing understanding of the relevance of APM component SNPs in HPV-mediated cervical carcinogenesis, may be an important determinant in design and effectiveness of HPV-targeted vaccines and immunotherapeutic regimens.

## References

[CR1] Barrett JC, Fry B, Maller J, Daly MJ (2005). Haploview: analysis and visualization of LD and haplotype maps. Bioinformatics.

[CR2] Becker T, Knapp M (2004). A powerful strategy to account for multiple testing in the context of haplotype analysis. Am J Hum Genet.

[CR3] Becker T, Cichon S, Jonson E, Knapp M (2005). Multiple testing in the context of haplotype analysis revisited: application to case-control data. Ann Hum Genet.

[CR4] Becker T, Schumacher J, Cichon S, Baur MP, Knapp M (2005). Haplotype interaction analysis of unlinked regions. Genet Epidemiol.

[CR5] Cao B, Tian X, Li Y, Jiang P, Ning T, Xing H, Zhao Y, Zhang C, Shi X, Chen D, Shen Y, Ke Y (2005). LMP7/TAP2 gene polymorphisms and HPV infection in esophageal carcinoma patients from a high incidence area in China. Carcinogenesis.

[CR6] Chang CC, Ferrone S (2007). Immune selective pressure and HLA class I antigen defects in malignant lesions. Cancer Immunol Immunother.

[CR7] Chow RA, Caeiro JL, Chen SJ, Garcia-Bertrand RL, Herrera RJ (2005). Genetic characterization of four Austronesian-speaking populations. J Hum Genet.

[CR8] Facoetti A, Nano R, Zelini P, Morbini P, Benericetti E, Ceroni M, Campoli M, Ferrone S (2005). Human leukocyte antigen and antigen processing machinery component defects in astrocytic tumors. Clin Cancer Res.

[CR9] Falk K, Rotzschke O (2002). The final cut: how ERAP1 trims MHC ligands to size. Nat Immunol.

[CR10] Hammer GE, Gonzalez F, Champsaur M, Cado D, Shastri N (2006). The aminopeptidase ERAAP shapes the peptide repertoire displayed by major histocompatibility complex class I molecules. Nat Immunol.

[CR11] Hanley JA (2001). A heuristic approach to the formulas for population attributable fraction. J Epidemiol Community Health.

[CR12] Heemels MT, Ploegh H (1995). Generation, translocation, and presentation of MHC class I-restricted peptides. Annu Rev Biochem.

[CR13] Hennig G, Gehrmann M, Stropp U, Brauch H, Fritz P, Eichelbaum M, Schwab M, Schroth W (2010). Automated extraction of DNA and RNA from a single formalin-fixed paraffin-embedded tissue section for analysis of both single-nucleotide polymorphisms and mRNA expression. Clin Chem.

[CR14] Kaufman J (2013). Antigen processing and presentation: evolution from a bird's eye view. Mol Immunol.

[CR15] Livak KJ (1999). Allelic discrimination using fluorogenic probes and the 5' nuclease assay. Genet Anal.

[CR16] Mehta AM, Jordanova ES, van Wezel T, Uh HW, Corver WE, Kwappenberg KM, Verduijn W, Kenter GG, van der Burg SH, Fleuren GJ (2007). Genetic variation of antigen processing machinery components and association with cervical carcinoma. Genes Chromosom Cancer.

[CR17] Mehta AM, Jordanova ES, Kenter GG, Ferrone S, Fleuren GJ (2008). Association of antigen processing machinery and HLA class I defects with clinicopathological outcome in cervical carcinoma. Cancer Immunol Immunother.

[CR18] Mehta AM, Jordanova ES, Corver WE, van Wezel T, Uh HW, Kenter GG, Fleuren GJ (2009). Single nucleotide polymorphisms in antigen processing machinery component ERAP1 significantly associate with clinical outcome in cervical carcinoma. Genes Chromosom Cancer.

[CR19] Meissner M, Reichert TE, Kunkel M, Gooding W, Whiteside TL, Ferrone S, Seliger B (2005). Defects in the human leukocyte antigen class I antigen processing machinery in head and neck squamous cell carcinoma: association with clinical outcome. Clin Cancer Res.

[CR20] Miettinen OS (1974). Proportion of disease caused or prevented by a given exposure, trait or intervention. Am J Epidemiol.

[CR21] Saric T, Chang SC, Hattori A, York IA, Markant S, Rock KL, Tsujimoto M, Goldberg AL (2002). An IFN-gamma-induced aminopeptidase in the ER, ERAP1, trims precursors to MHC class I-presented peptides. Nat Immunol.

[CR22] Sasieni PD (1997). From genotypes to genes: doubling the sample size. Biometrics.

[CR23] Saveanu L, Carroll O, Lindo V, Del Val M, Lopez D, Lepelletier Y, Greer F, Schomburg L, Fruci D, Niedermann G, van Endert PM (2005). Concerted peptide trimming by human ERAP1 and ERAP2 aminopeptidase complexes in the endoplasmic reticulum. Nat Immunol.

[CR24] Seliger B, Ritz U, Ferrone S (2006). Molecular mechanisms of HLA class I antigen abnormalities following viral infection and transformation. Int J Cancer.

[CR25] Shepard EM, Chow RA, Suafo'a E, Addison D, Perez-Miranda AM, Garcia-Bertrand RL, Herrera RJ (2005). Autosomal STR variation in five Austronesian populations. Hum Biol.

[CR26] Vet JN, de Boer MA, van den Akker BE, Siregar B, Lisnawati, Budiningsih S, Tyasmorowati D, Moestikaningsih, Cornain S, Peters AA, Fleuren GJ (2008). Prevalence of human papillomavirus in Indonesia: a population-based study in three regions. Br J Cancer.

[CR27] Yan J, Parekh VV, Mendez-Fernandez Y, Olivares-Villagomez D, Dragovic S, Hill T, Roopenian DC, Joyce S, Van Kaer L (2006). In vivo role of ER-associated peptidase activity in tailoring peptides for presentation by MHC class Ia and class Ib molecules. J Exp Med.

[CR28] York IA, Chang SC, Saric T, Keys JA, Favreau JM, Goldberg AL, Rock KL (2002). The ER aminopeptidase ERAP1 enhances or limits antigen presentation by trimming epitopes to 8-9 residues. Nat Immunol.

[CR29] York IA, Brehm MA, Zendzian S, Towne CF, Rock KL (2006) Endoplasmic reticulum aminopeptidase 1 (ERAP1) trims MHC class I-presented peptides in vivo and plays an important role in immunodominance. Proc Natl Acad Sci USA 103:9202–9207 10.1073/pnas.0603095103 PMC1482590 16754858

